# Assessment of auditory and vestibular function and gene therapy in the Snell’s waltzer mouse model of human deafness and balance dysfunction

**DOI:** 10.1007/s00335-026-10263-y

**Published:** 2026-08-28

**Authors:** Eyal Marton, Roni Hahn, Lior Bikovski, Gwenaëlle S. G. Géléoc, Jeffrey R. Holt, Matti Mintz, Karen B. Avraham

**Affiliations:** 1 Department of Human Genetics & Computational Medicine, Gray Faculty of Medical & Health Sciences and Sagol School of Neuroscience, Tel Aviv University, 6997801 Tel Aviv, Israel; 2 School of Psychological Sciences and Sagol School of Neuroscience, Tel Aviv University, 6997801 Tel Aviv, Israel; 3 Myers Neuro-Behavioral Core Facility, Gray Faculty of Medical & Health Sciences, Tel Aviv University, 6997801 Tel Aviv, Israel; 4 Department of Otolaryngology & Neurology, F.M. Kirby Neurobiology Center, Boston Children’s Hospital, Harvard Medical School, Boston, MA 02115 USA

## Abstract

The Snell’s waltzer mouse (*Myo6*^*sv/sv*^) serves as a model for human deafness and vestibular behavioral impairment, caused by a spontaneous 130 bp recessive deletion in the *Myo6* gene. In this study, we characterized the auditory and vestibular phenotypes of *Myo6*^*sv/sv*^ mice. These mice exhibit profound hearing loss, with cochlear hair cell stereocilia beginning to fuse soon after birth, ultimately leading to disorganization of hair bundles and degeneration of hair cells. Mice also exhibit behavioral phenotypes characterized by severe imbalance, hyperactivity with bouts of circling, and delayed spatial learning of a novel environment, but preserved normal behavioral circadian rhythms. These behaviors emerge in association with the loss of the characteristic staircase morphology of vestibular hair cell stereocilia soon after birth and the subsequent profound elongation of the stereocilia. Adeno-associated virus (AAV) gene replacement therapy, delivered on the day of birth or one day after, failed to restore auditory or vestibular function. Our findings underscore the essential role of *Myo6* in the auditory and vestibular systems and imply prenatal intervention may be required for effective therapy.

**Supplementary Information:**

The online version contains supplementary material available at https://doi.org/10.1007/s00335-026-10263-y.

## Introduction

Humans rely on a variety of sensory systems, including hearing, vision, vestibular, and proprioception, to navigate and perform daily activities. Auditory and vestibular perception are especially critical. The auditory system facilitates communication and awareness of the surroundings. The vestibular system, used as the inertial guidance system, allows maintenance of a balanced upright posture, keeping eyes focused on a target when the head moves, and distinguishing between self vs. environmental motion (Kandel et al. 2000).

According to the World Health Organization (WHO), over 5% of the world’s population requires rehabilitation to address their disabling hearing loss. By 2050, one in every ten people will have disabling hearing loss (World Health Organization 2025). Hearing loss is associated with lower performance in cognitive tasks such as spatial orientation and learning (Avci and Cicek Cinar 2024) and ultimately with an increased risk of dementia (Powell et al. 2021). Vestibular impairment also has severe consequences, such as imbalance (Antoine et al. 2017), gaze instability (Christy 2018), and anxiety (Balaban et al. 2011).

While hearing loss and vestibular impairment may be triggered by drug or noise exposure, or pathological conditions such as labyrinthitis or vestibular schwannoma, they are often the result of pathogenic genetic variants. According to the Deafness Variation Database, DVD v9.2, 224 genes are involved in non-syndromic hearing loss, some of which are also associated with vestibular dysfunction (Azaiez et al. 2018; Deafness Variation Database 2026). One of these genes, *MYO6*, encodes the protein myosin VI, a molecular motor necessary for maintaining the structural integrity of sensory hair cells (Avraham et al. 1995). Pathogenic variants in *MYO6* contribute to dominant (Melchionda et al. 2001) and recessive (Ahmed et al. 2003) forms of hearing loss in humans.

Although studies on vestibular dysfunction in humans are limited, mouse models provide valuable insights into the underlying mechanisms of these impairments. For example, studies in mouse models of vestibular dysfunction reveal secondary effects such as hyperactivity and anxiety-like behavior (Avni et al. 2009), reduced social interaction (Zheng et al. 2008), delay in sensorimotor development, and spatial orientation dysfunction (Le Gall et al. 2019). In addition, mouse models of hearing loss and vestibular impairments offer a platform to explore potential therapeutic interventions (Hahn and Avraham 2023).

The Snell’s waltzer mouse (*Myo6*^*sv/sv*^) is a model for both human deafness and vestibular impairment (Deol and Green 1966). The mutation is a spontaneous recessive 130 bp deletion in the *Myo6* gene, predicted to cause a frameshift that introduces a stop codon at the beginning of the protein’s neck region (Avraham et al. 1995). Although myosin VI has been extensively studied, the impact of its mutations on vestibular function has been mostly descriptive, the exception being a single study in which the vestibular deficit in Snell’s waltzer mice was confirmed by absence of the linear vestibular-evoked potentials (VsEPs), indicating a gravity receptor deficit (Jones et al. 2005). In this report, we characterize both the auditory and vestibular phenotypes of Snell’s waltzer mice and its secondary effects.

In addition, we used a posterior semi-circular canal or utricle injection of an adeno-associated virus (AAV) construct encoding *Myo6* to attempt to rescue hearing and vestibular function compromised by the mutation. Our results show that injection at postnatal day 0 (P0) failed to restore function and suggest that *Myo6* gene therapy may have to be provided before birth in mice.

## Materials and methods

### Animals

All procedures involving animals were performed in accordance with the regulations and the guidelines set forth by the National Institutes of Health Guide for the Care and Use of Laboratory Animals. Protocols were approved by the Animal Care and Use Committee at Tel Aviv University (01–21-063 and TAU-MD-IL 2507-140-4).

B6 x STOCK *Tyr*^*c−ch*^ *Bmp5*^*se*^ +/+ *Myo6*^*sv*^/J mice (RRID: IMSR_JAX:000578) were obtained from the Jackson Laboratory (JAX, USA). Upon arrival, mice were housed in the vivarium at Tel Aviv University under a 12 h light/dark cycle (lights on between 7:00 and 19:00), at a controlled temperature of 25–28°C and provided with *ad libitum* access to food and water. Backcrossing, rederivation and maintenance were carried out using the standard C57BL/6J line, with a backcross and intercross routine, ensuring background consistency across experimental cohorts. Once the colony was established, mice intended for gene therapy were continuously kept under this regular light/dark cycle, and injections and behavioral tests were performed during the mice’s inactive phase of the cycle. Mice intended for phenotype characterization were switched to a reversed 12 h light/dark cycle (lights on between 20:00 and 8:00), and behavioral tests were performed in the mice’s active phase of the cycle under red lights in the following order: swim test, rotarod test, 15 min open field test, 1 h open field test, PhenoTyper home-cage monitoring, and lastly auditory brain response (ABR), with a day interval between tests. All tests used a balanced mix of both males and females. All *n* values represent the number of individual mice used in each experiment.

### Gene therapy

#### Plasmid preparation and AAV production

An AAV-Myo6 plasmid was designed and sent for production in VectorBuilder (VectorBuilder Inc., USA). The plasmid contained the following: AAV2 ITR, a CMV promoter, Kozak translation initiation sequence, the correct coding sequence of *Myo6* (ENSMUST00000035889_15), poly(A) signal, and AAV2 ITR, in the respective order.

The NucleoBond Xtra Maxi Plus DNA extraction kit was used to isolate DNA. AAV production was carried out by the Viral Core at Boston Children’s Hospital’s Viral Vector Core, using the AAV2/9-PHP.B capsid (Lee et al. 2020). Two versions of the AAV, one with a CMV promoter and the second with a CB6 promoter containing an HA tag, were produced. The titer of the AAV2/9-PHP.B.CMV.Myo6 was 5.00 E^+ 13^ gc/ml, and the titer of the AAV2/9-PHP.B.CB6.Myo6-HA was 2.86 E^+ 13^ gc/ml. Vectors were aliquoted into 10 µL vials and stored at −80°C. HeLa cells were transfected with the plasmid and the AAV, and immunofluorescence was performed to validate myosin VI expression.

#### AAV injections

 At P0, mice were anesthetized by induced hypothermia, and the entire surgery was performed on an ice block under a binocular microscope. Using small spring scissors, a 1–2 mm incision was made in the skin 1–2 mm caudal to the base of the left outer ear. Soft tissue was then carefully dissected to expose the posterior semi-circular canal and utricle. Either of the two was gently punctured with a glass micropipette (Drummond, Broomall, PA 2-000-100) with a fine tapered tip (Sutter Instrument, Novato, CA, USA). The micropipette was stabilized on a stereotaxic arm, and 1.2 µL of the AAV solution was injected by a microdialysis pump (CMA/102, Sweden) at a slow 10 nl/s rate (Taiber et al. 2021). The incision was sutured with a single stitch, and 5% lidocaine cream was applied for pain relief. After the surgery, mice were placed on a heating pad, cleaned thoroughly with 70% ethanol, and returned to their mother’s cage within 1.5 h of separation.

### Images of the inner ear

#### Confocal

Mice were sacrificed by CO_2_ inhalation followed by decapitation. The inner ear was dissected and immersed into 2 ml of 4% paraformaldehyde (PFA) for overnight fixation at 4 °C. Cochlea and utricle were dissected into phosphate-buffered saline (PBS) the following day. Samples were permeabilized and blocked in 2% Triton X-100 and 10% normal goat serum for 1 h at room temperature and then incubated overnight with rabbit polyclonal myosin VIIa (Proteus Biosciences) or rabbit polyclonal myosin VI (Proteus Biosciences) or an anti-HA tag antibody (ab9110, 1:250) diluted in phosphate green antibody diluent (Bar Naor, Ltd., Israel). Samples were incubated with secondary goat anti-rabbit Alexa Fluor 568 antibody at 1:250, DAPI (4′,6-diamidino-2-phenylindole) at 1:500, and Phalloidin 488 at 1:250, diluted in PBS, and then mounted in ProLong Gold Antifade Mountant (Thermo Fisher Scientific) and imaged using a Zeiss LSM 880 (Zeiss, Oberkochen, Germany) equipped with an Airyscan detector.

#### Scanning electron microscopy (SEM)

Mice were sacrificed by decapitation at P0 and P3 or by CO_2_ inhalation followed by decapitation at P30. The inner ear was dissected and fixed in 2 ml of 2.5% glutaraldehyde for 1 h at 4°C on a shaker, then transferred to 2 ml of PBS at 4°C. Cochlea, utricle, and semicircular canals were dissected in PBS three days later. Samples were then moved to 4-well dishes and a hydration protocol of rinse with 1% osmium tetroxide (OsO₄) in PBS for 1 h, followed by two cycles of rinse with osmium for 20 min, 1 h rinse with 1% osmium in double-distilled water (DDW), 5 min rinse with 30% ethanol, 5 min rinse with 50% ethanol, and rinse with 70% ethanol. Samples were stored at 4^**o**^C overnight and rinsed with 100% ethanol the following day. Ethanol was evaporated with liquid CO_2_, and then the CO_2_ was evaporated. Images were obtained using a Zeiss Gemini SEM 300 high-resolution field emission SEM (HRSEM) with an accelerating voltage 10.0 kV at the Center for Nanoscience and Nanotechnology at Tel Aviv University.

#### Measurement of hair cell stereocilia length

Stereocilia length was assessed in hair cells from the cochlear inner hair cell (IHC) middle region and vestibular central striolar zone using 92.26 μm thick confocal z-stacks of tissue stained with phalloidin, myosin VIIa, and 4′,6-diamidino-2-phenylindole (DAPI). Using Fiji-ImageJ software, the length was calculated as the mean of the three longest stereocilia, starting from the base up to the tip of each stereocilium.

### Tests of hearing and balance functions

#### Auditory brainstem response (ABR) and distortion-product otoacoustic emission (DPOAE)

Mice were anesthetized by intraperitoneal injection of 0.01 ml/g of a mix (2 mL mix of 1.7 mL of PBS, 0.2 mL of ketamine, and 0.1 mL of xylazine), checked for pain reflex by toe pinching, and given a subcutaneous boost if responsive. Recordings were conducted in an acoustic chamber (MAC-1, Industrial Acoustic Company, Naperville, IL, USA). Three subdermal electrodes were applied: a reference electrode below the checked ear, a ground electrode below the other ear, and an active electrode over the vertex. For ABR recordings, mice were placed on a heating pad facing a microphone 10 cm away, with the tested ear 5 cm away from a speaker. Mice were then presented with pure tones at frequencies of 6, 12, 18, 24, 30, and 35 kHz, at intensities ranging from 10 to 90 dB SPL, in 5 dB increments. ABR recordings were sampled at 500 Hz and averaged over 500 tones. The hearing threshold was the lowest sound intensity that triggered waves 1 to 5. For DPOAE recordings, a microphone attached to two MF-1 speakers presenting two primary tones (f1 and f2) at a frequency ratio of 1.2, was placed inside the mouse’s ear canal. Each frequency–tone combination was averaged over 256 samples. The amplitude of the distortion product at a frequency of 2f1–f2 and the surrounding average noise level were extracted from the averaged responses using a designated R algorithm (Rstudio, Boston, MA; (Taiber et al. 2021).

#### Swim test

Mice were held by their tails and dropped into a round transparent glass beaker filled with 25°C water (Avni et al. 2009). The performance was scored as ‘success’ if the mice quickly resurfaced and swam toward the periphery of the beaker; or ‘fail’ if they failed to resurface for more than 5 s and had to be rescued. At the end of the procedure, the mice were dried and placed in a separate cage on a heating pad.

#### Rotarod test

Mice were tested on eight RotaRod (Ugo Basile, Italy) trials, interspaced by a 5 min resting period (Tung et al. 2014). On each trial, mice were placed on a 3.2 cm diameter rod that revolved at 4 rpm and accelerated to 50 rpm over 300 s. The performance was scored as a time to fall (TTF) with a maximum of 300 s.

#### Open field test

Mice were placed in the corner of the 50 by 50 cm, white Plexiglas open field, facing the arena center. Mice were video recorded for either 15 min or 1 h using a ceiling camera. Videos were sampled offline at 60 Hz, X-Y coordinates were extracted (EthoVision XT 17, Noldus Information Technology, The Netherlands), and the travel path and heatmap of the mice were generated using RStudio. The behavior was scored as traveled distance, number of clockwise and counterclockwise full-body rotations, and time spent in the arena’s center (25 by 25 cm).

#### PhenoTyper home-cage monitoring system

Mice were housed individually, starting at 8:00 am, in a 30 by 30 cm PhenoTyper cage system (PhenoTyper 2.0, Noldus Information Technology, The Netherlands) for three days. The cages, with transparent plexiglass walls and an opaque white plexiglass floor, were equipped with a feeding station, a water bottle, and a 10 by 10 cm shelter. Food and water were provided ad libitum. The cage’s lid is equipped with an infrared-sensitive camera for video tracking. Mice were allowed to move freely inside the cage without any human handling. EthoVision was used as video tracking and trial control software (EthoVision XT 17,). The behavior was scored as the travelled distance (Tseitlin et al. 2023).

### Tests procedures

#### Phenotype characterization

Mice intended for phenotype characterization were spared the inner ear injection and were subjected to behavioral tests at the 4th week of age (P22-P28) in the following order: swim, rotarod, open field, and Phenotyper home cage monitoring. All tests were carried out during the mice’s daily active phase under red illumination at random hours to reduce habituation, with a day interval between behavioral tests to reduce stress. At P30, ABR and DPOAE were performed, and mice were sacrificed immediately after for confocal or SEM imaging of hair cell morphology.

#### Phenotype after gene therapy

Wild-type and *Myo6*^*sv/sv*^ mice were injected with AAV.CMV.Myo6 or AAV.CB6.Myo6. Behavioral tests were conducted in mice at the 4th week of age (P22-P28) in the following order: swim, rotarod. All tests were carried out during the mice’s inactive phase under white illumination with a day interval between behavioral tests. At P30, ABR and DPOAE tests were performed, and mice were sacrificed immediately after for confocal imaging of hair cell morphology and validation of AAV expression.

### Statistics

**Fig. 1:**
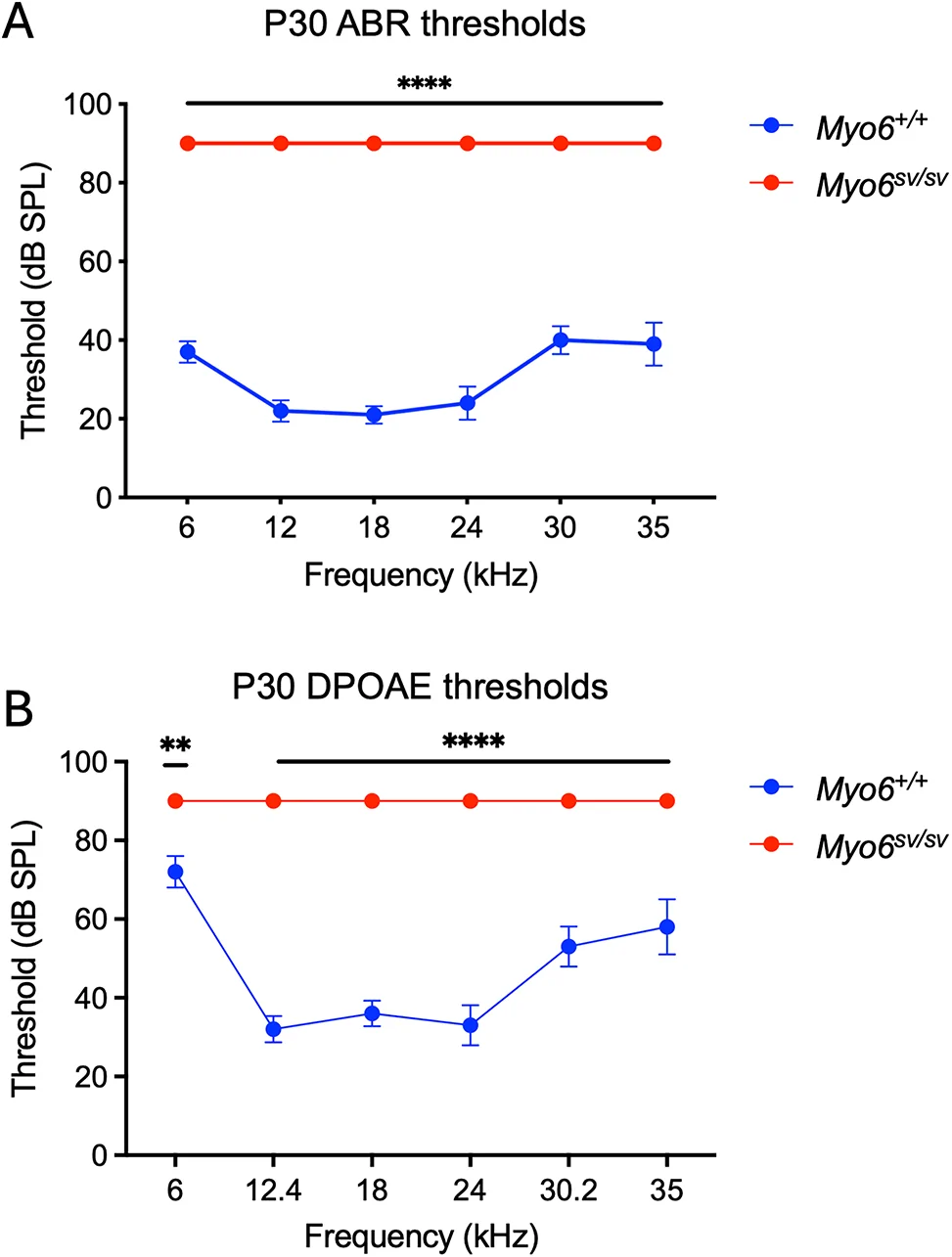
Snell’s waltzer mice demonstrate a profound hearing loss. **A** ABR threshold (mean ± SD) at P30 of *Myo6*^*+/+*^ (*n* = 7; males = 4, females = 3) and *Myo6*^*sv/sv*^ (*n* = 9; males = 5, females = 4). **** = *p* < 0.0001. **B** DPOAE threshold (mean ± SD) at P30 of *Myo6*^*+/+*^ (*n* = 5; males = 4, female = 1) and *Myo6*^*sv/sv*^ (*n* = 5; males = 3, female = 2). *n* = number of mice.

**Fig. 2:**
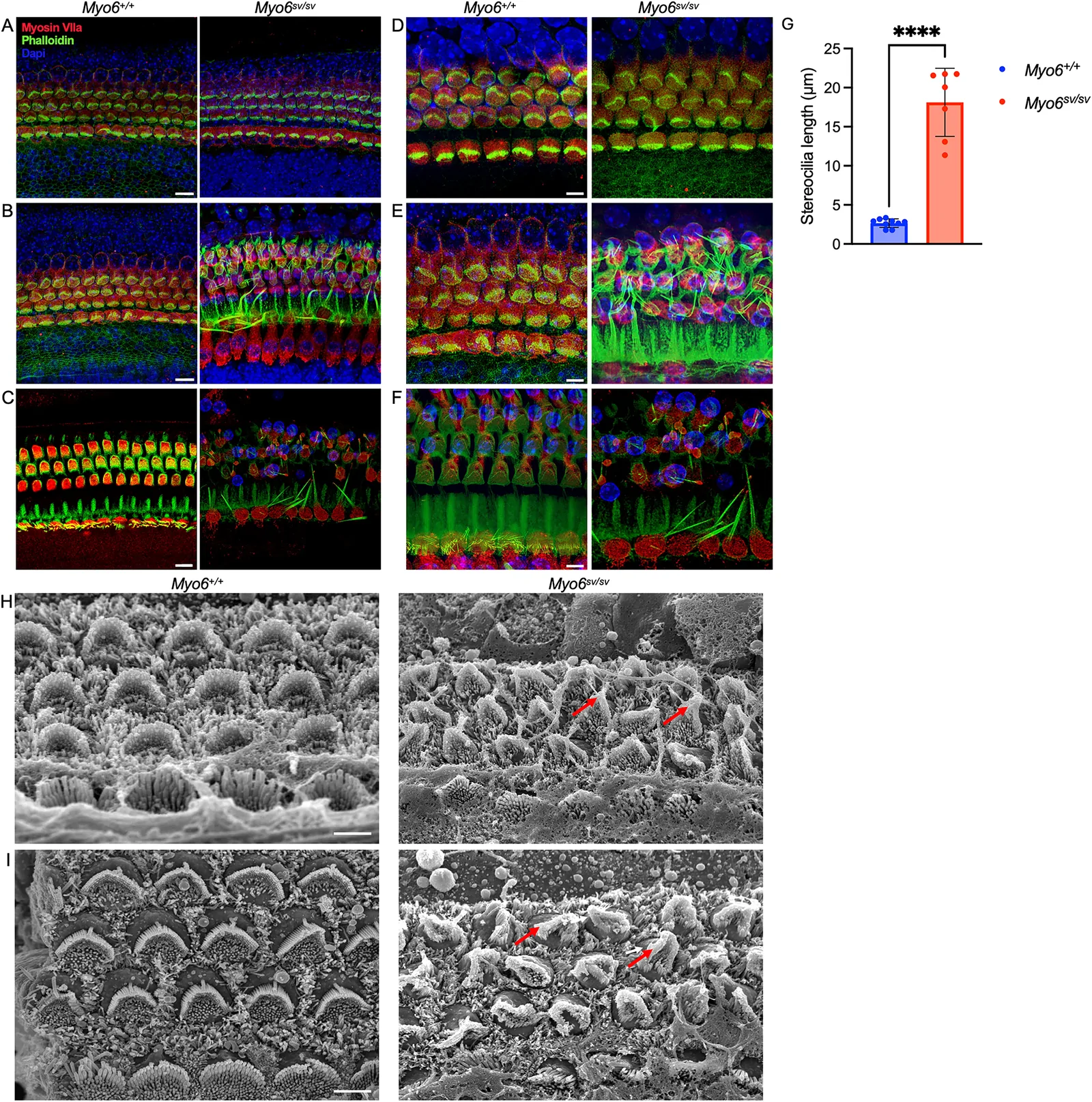
Mutation in the *Myo6* gene leads to fused stereocilia in the cochlea at early stages, followed by loss of OHC ‘v’ shape and staircase structure and almost complete hair cell loss at P30.** A–C** Representative X2 zoomed confocal images of the middle region of *Myo6*^*+/+*^ and *Myo6*^*sv/sv*^ cochleae labeled with antibodies for hair cell marker myosin VIIa (red), phalloidin (green), and DAPI (blue), at P3 (**A**), P7 (**B**), and P30 (**C**). **D–F** Representative X4 zoomed confocal images of the middle region of *Myo6*^*+/+*^ and *Myo6*^*sv/sv*^ cochleae labeled with antibodies for myosin VIIa (red), phalloidin (green), and DAPI (blue), at P3 (**D**), P7 (**E**), and P30 (**F**). **G** Cochlear hair cell stereocilia length (mean ± SD) at P30 of *Myo6*^*+/+*^ (*n* = 9) and *Myo6*^*sv/sv*^ (*n* = 7). n = number of mice. ****p < 0.0001. **H–I** Representative SEM images of the middle region of the cochlea OHC of *Myo6*^*+/+*^ (**H**) and *Myo6*^*sv/sv*^ (**I**) at P0 and P3, with fusion shown by red arrows. Scale bars: **A**–**C**, 10 μm; **G**–**F**, 5 μm; **H**–**I**, 3 μm

**Fig. 3:**
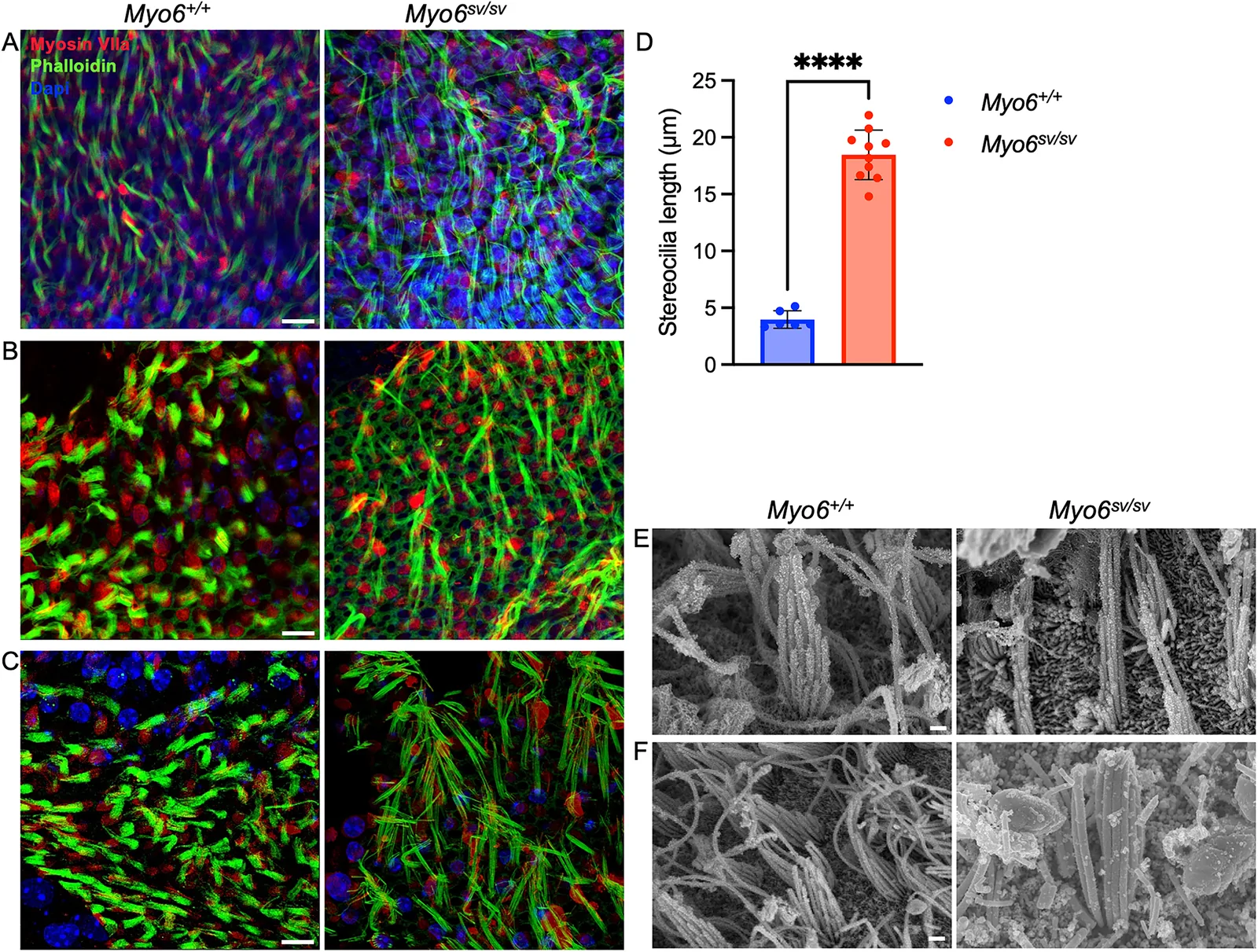
Utricular hair cells lose their staircase structure at the early stage of development and have elongated stereocilia at later stages.** A–C** Representative confocal images of zoomed-in utricles of *Myo6*^*+/+*^ and *Myo6*^*sv/sv*^, labeled with antibodies for myosin VIIa (red), phalloidin (green), and DAPI (blue), at P0 (**A**), P3 (**B**) and P30 (**C**). **D** Utricle hair cells’ stereocilia length (mean ± SD) at P30 of *Myo6*^*+/+*^ (*n* = 6) and *Myo6*^*sv/sv*^ (*n* = 9). *n* = number of mice. **** *p* < 0.0001. **E–F** Representative SEM images of a utricular hair cell of *Myo6*^*+/+*^ and *Myo6*^*sv/sv*^ at P0 (**E**) and P3 (**F**). Scale bars: **A**–**C**, 5 μm; **E**–**F**, 1 μm

**Fig. 4:**
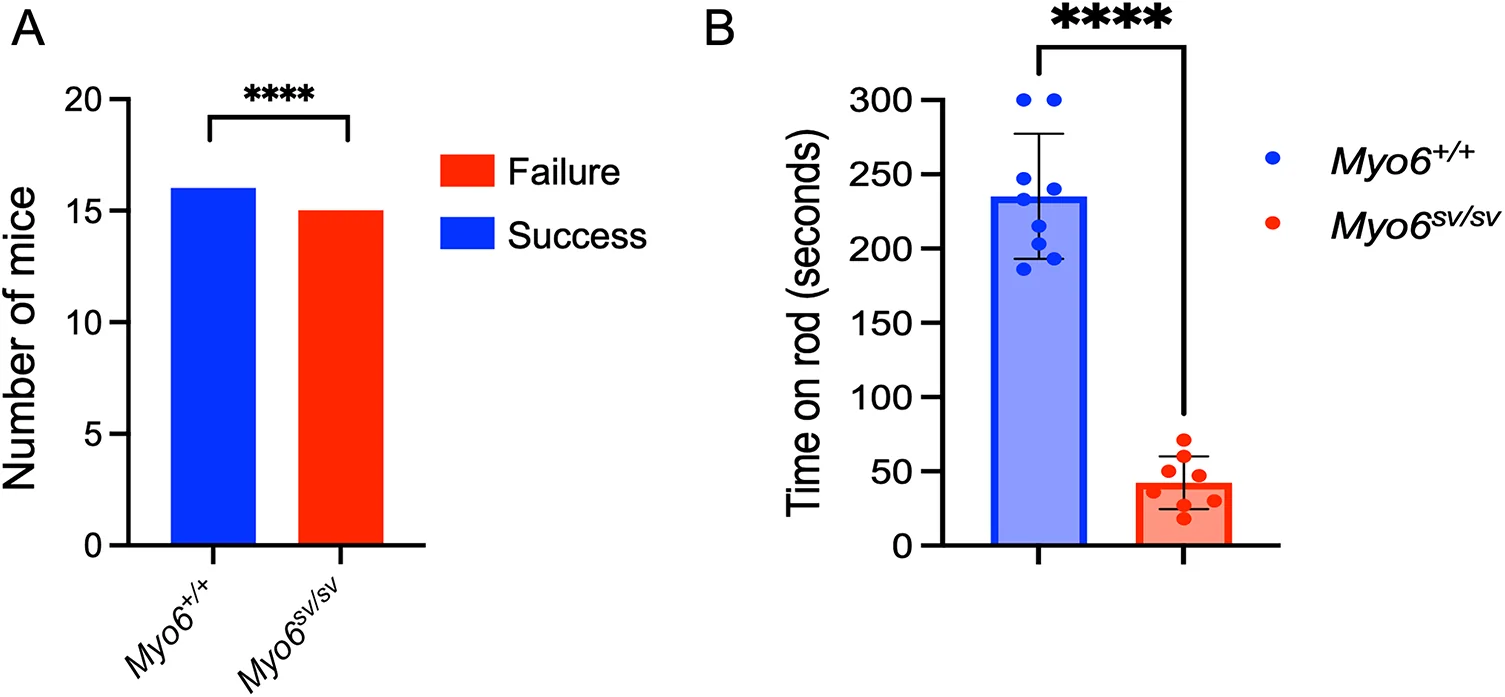
Impaired vestibular behavior of *Myo6*^*sv/sv*^ mice. **A** Swim test performance at P30 of *Myo6*^*+/+*^ (*n* = 16; males = 8, females = 8) and *Myo6*^*sv/sv*^ (*n* = 15; males = 8, females = 7). ****p < 0.0001. **B** Time spent on the rotarod (mean ± SD) at P30 of *Myo6*^*+/+*^ (*n* = 9; males = 5, females = 4) and *Myo6*^*sv/sv*^ (*n* = 8; males = 4, females = 4). *n* = number of mice. ****p < 0.0001

**Fig. 5:**
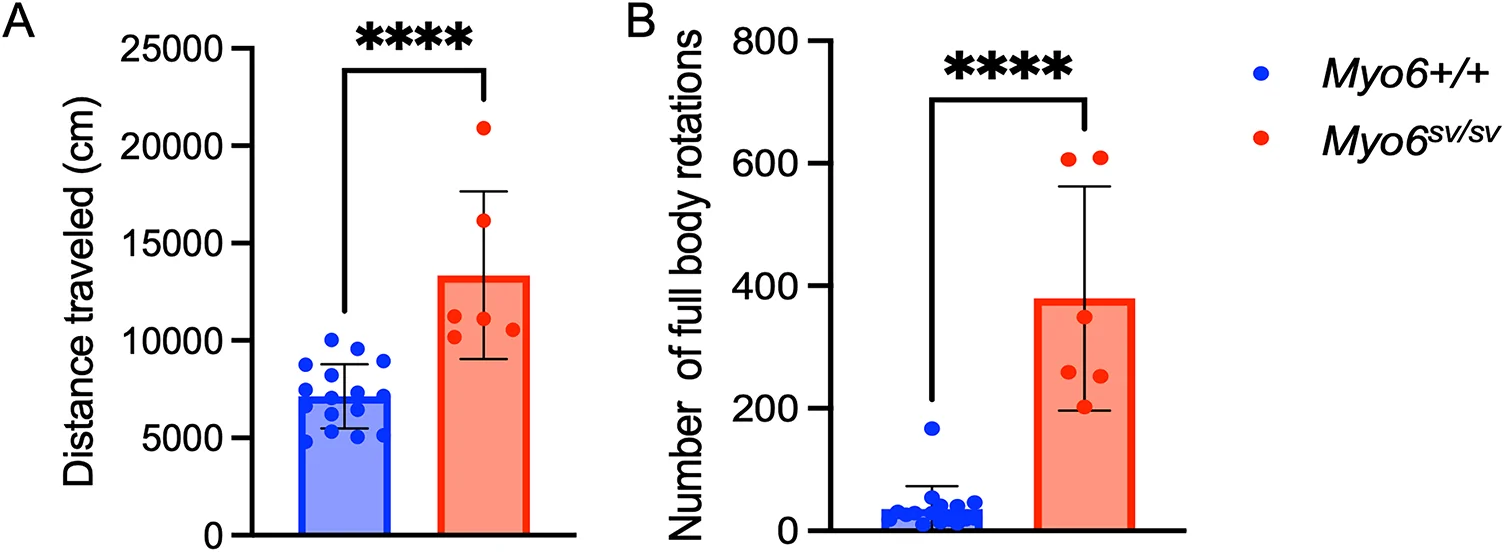
*Myo6*^*sv/sv*^ exhibit hyperactivity and circling behavior. **A** Distance travelled (mean ± SD) at P30 in the 15 min open-field test of *Myo6*^*+/+*^ (*n* = 16; males = 8, females = 8) and *Myo6*^*sv/sv*^ (*n* = 6; males = 3, females = 3) mice. **B** Number of full body rotations (mean ± SD) at P30 in the 15 min open -field test of *Myo6*^*+/+*^ (*n* = 16; males = 8, females = 8) and *Myo6*^*sv/sv*^ (*n* = 6; males = 3, females = 3) mice. *n* = number of mice. ****p < 0.0001

**Fig. 6:**
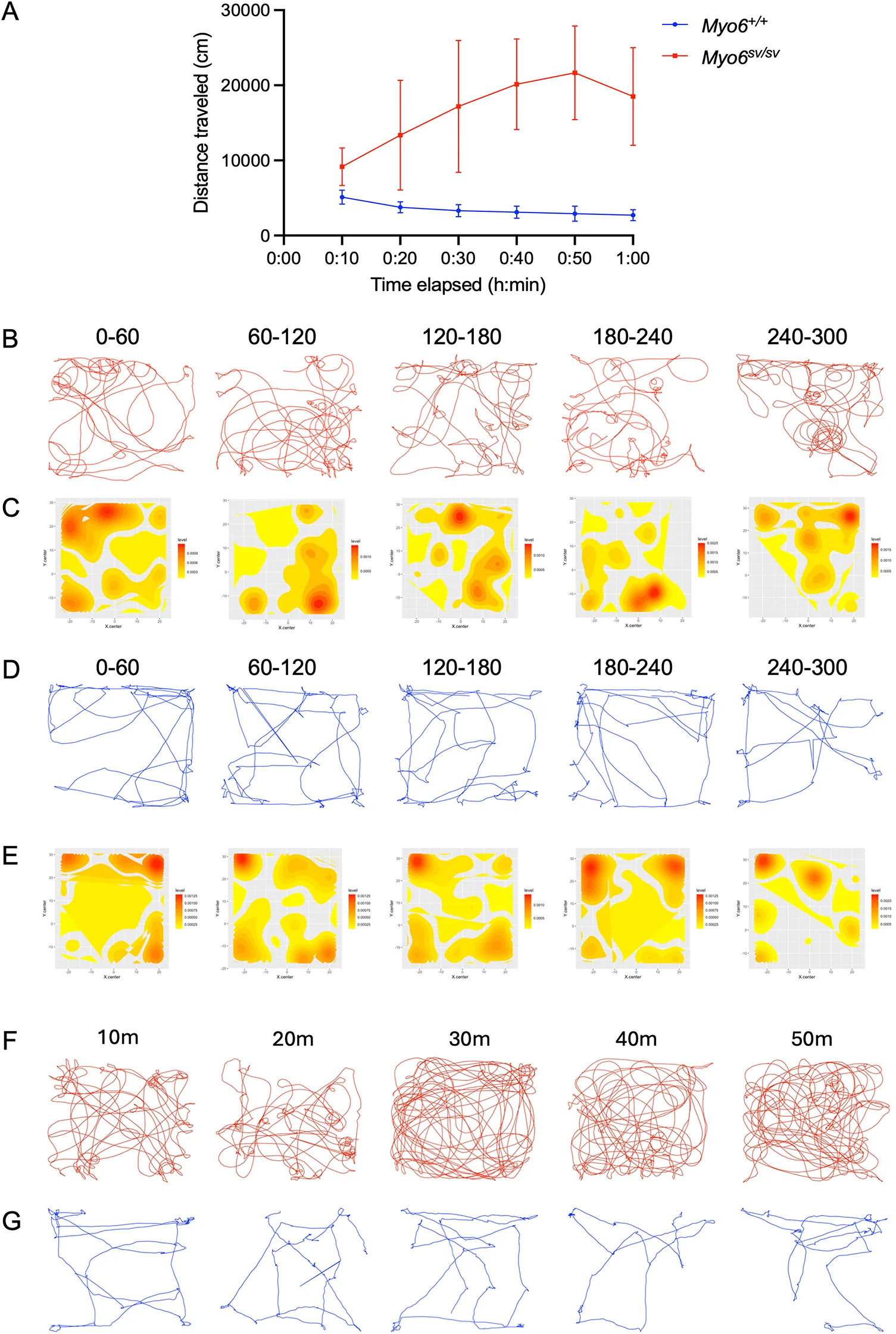
*Myo6*^*sv/sv*^ exhibit exploratory behavior with an increase in activity over time in the 60-min long open field test. **A** Travelled distance across 10 min intervals (mean ± SD) of *Myo6*^*+/+*^ (*n* = 5; males = 3, females = 2) and *Myo6*^*sv/sv*^ (*n* = 7; males = 4, females = 3) mice at P30 in a 60 min-long open field test. **B–E** Travel path and travel path heatmap of representative *Myo6*^*sv/sv*^ (**B–C)** and *Myo6*^*+/+*^ (**D–E**) mice presented for 1-min periods across the first 5 min of the open field test. **F–G** Travel path of representative *Myo6*^*sv/sv*^ (**F**) and *Myo6*^*+/+*^ (**G**) mice presented for 1-min periods at the 10th, 20th, 30th, 40th, and 50th min of the open field test. *n* = number of mice. The color coding shown in the legend in panel A also applies to panels B, D, F, and G

**Fig. 7:**
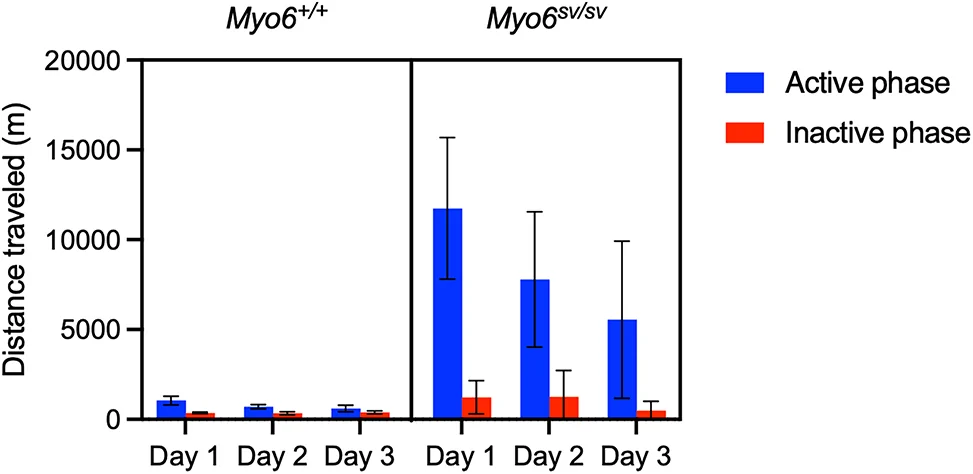
*Myo6*^*sv/sv*^ mouse hyperactivity is modulated by normal circadian rhythm and long-term habituation to familiar space. Daily travelled distance (mean ± SD during active (night) and inactive (light) daily phase of *Myo6*^*+/+*^ (*n* = 6; males = 3, females = 3) and *Myo6*^*sv/sv*^ (*n* = 10; males = 5; females = 5) mice in the 3-day-long PhenoTyper home-cage test at P30. *n* = number of mice.

**Fig. 8:**
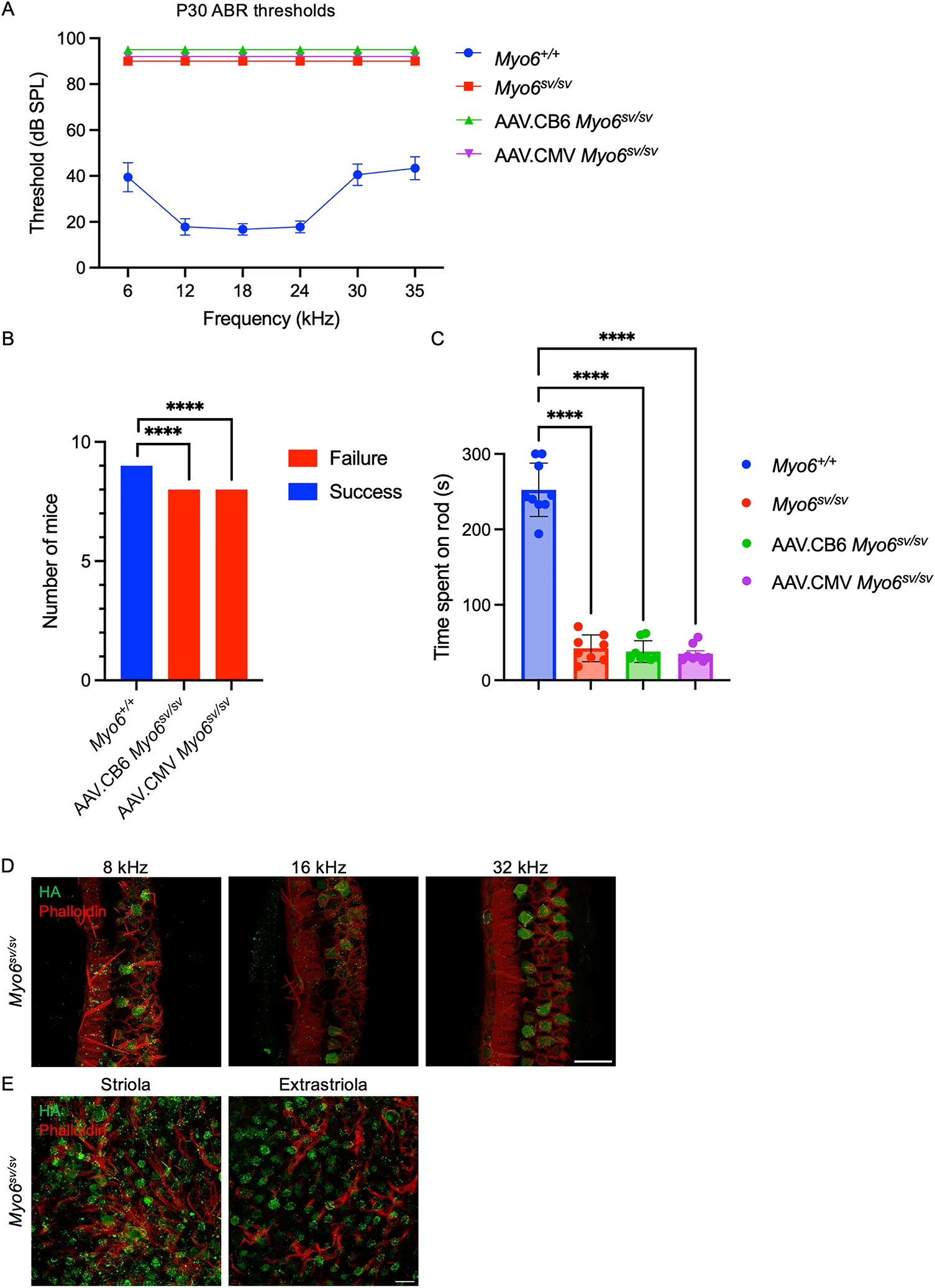
Auditory and vestibular function following AAV transduction in inner ears.** A** ABR threshold (mean ± SD) at P30 of *Myo6*^*+/+*^ (*n* = 9; males = 5, females = 4) and *Myo6*^*sv/sv*^ (*n* = 8; males = 4, females = 4), AAV.CB6 *Myo6*^*sv/sv*^ (*n* = 8; males = 4, females = 4), AAV.CMV *Myo6*^*sv/sv*^ (*n* = 8; males = 4, females = 4). **** = *p* < 0.0001. **B** Swim test performance at P30 of *Myo6*^*+/+*^ (*n* = 9; males = 5, females = 4), AAV.CB6 *Myo6*^*sv/sv*^ (*n* = 8; males = 4, females = 4) and AAV.CMV *Myo6*^*sv/sv*^ (n = 8; males = 4, females = 4). **** *p* < 0.0001. **C** Time spent on the rotarod (mean ± SD) at P30 of *Myo6*^*sv/sv*^ (*n* = 9; males = 5, females = 4), *Myo6*^*sv/sv*^ (*n* = 8; males = 4, females = 4), AAV.CB6 *Myo6*^*sv/sv*^ (*n* = 8; males = 4, females = 4) and AAV.CMV *Myo6*^*sv/sv*^ (*n* = 8; males = 4, females = 4). *n* = number of mice. **** *p* < 0.0001. **D** Efficient expression of HA-tagged *Myo6* CB6.AAV delivery does not restore morphology. Representative confocal images of cochlear hair cells from 8, 16, and 32 kHz regions of injected *Myo6*^*sv/sv*^ with CB6.AAV stained with antibodies for HA tag (green) and phalloidin (red). **E** Representative confocal images of vestibular hair cells from the utricle of injected *Myo6*^*sv/sv*^ stained with antibodies for HA tag (green) and phalloidin (red). Scale bars: 10 μm

All statistical analyses and graphs were performed and produced by Prism 11 software (GraphPad version 11.0.2, San Diego, CA). ABR and DPOAE data (Fig. 1) were analyzed by a two-way ANOVA. Stereocilia length (Figs. 2G and 3D), rotarod (Figs. 4B, 8C, S2B and S4C), and the 15-min open-field data (Figs. 5 and 6A) were analyzed using independent-samples *t*-tests. The 1-h open-field data (Fig. 6A) were analyzed using a two-way repeated-measures ANOVA, and the PhenoTyper home-cage data (Fig. 7) were analyzed using a three-way repeated-measures ANOVA. Swim test performance was analyzed using Fisher’s exact test. Data is presented as mean ± SD and figure legends detail sample sizes and P values. Heat maps and travel paths were produced by RStudio (Posit Software version 2025.09.1 + 401, Boston, MA). Randomization was used whenever applicable.

## Results

### Snell’s waltzer (*Myo6*^*sv/sv*^) mice display profound hearing loss

Auditory function of *Myo6*^*+/+*^ and *Myo6*^*sv/sv*^ mice was assessed at P30 by recording ABRs evoked by pure tones ranging from 6 to 35 kHz at 10 to 90 dB SPL (Fig. 1A). While *Myo6*^*+/+*^ mice demonstrated normal ABR thresholds (between 20 and 40 dB SPL), *Myo6*^*sv/sv*^ mice displayed no ABR responses to any tone at any intensity. Recordings of DPOAEs at P30 (Fig. 1B), which reflect the function of outer hair cells (OHC), also showed the absence of responses in the mutants. These results confirm the previous report that Snell’s waltzer mice suffer from profound hearing loss (Avraham et al. 1995; Deol and Green 1966).

Onset and severity of cochlear epithelium and sensory hair bundle damage in *Myo6*^*+/+*^ and *Myo6*^*sv/sv*^ mice were assessed by confocal and SEM imaging (Fig. 2). Immunostaining was performed in fixed tissues at P3, P7, and P30 using the hair cell marker, anti-Myo7a, along with phalloidin actin staining and DAPI. Representative confocal images from the middle region of the cochlea are shown in Fig. 2A–F. At P3, we did not observe any loss of hair cells. OHC and IHC hair bundles also appeared well preserved. By P7, some OHCs were lost, or their hair bundles exhibited mild disorganization. In contrast, IHCs displayed a single elongated fused bundle, although no hair cell loss was observed at this stage. By P30, partial OHC loss was observed along with severe disruption of the hair bundles, with similar but less pronounced changes observed in the IHCs. The changes were similar across the basal, middle, and apical regions of the cochlea at all ages examined (Fig. S1). At this stage, both IHCs and OHCs displayed a single elongated fused bundle, with the stereocilia being significantly longer in Snell’s waltzer (mean = 18.11 μm, SD = 4.35) than wild type (mean = 2.66 μm, SD = 0.5543) (t(14) = 10.65, *p* < 0.0001) (Fig. 2G). To further assess hair bundle morphology, we prepared and imaged P0 and P3 samples by SEM. SEM images of hair bundles at P0 revealed the presence of fused stereocilia and further disorganization by P3, with fused stereocilia and loss of the ‘v’-shaped structure in several cells (Fig. 2H–I).

### Snell’s waltzer mice display a balance deficit

Onset and severity of vestibular hair cell damage in *Myo6*^*+/+*^ and *Myo6*^*sv/sv*^ mice were assessed by confocal imaging and SEM (Fig. 3). Confocal imaging revealed an intact utricle with no apparent hair cell loss at P0, P3, and P30. (Fig. 3A–C), with the stereocilia being significantly longer in *Myo6*^*sv/sv*^ (mean = 18.46 μm, SD = 2.175) than wild type (mean = 3.97 μm, SD = 0.7646) (t(14) = 15.6, *p* < 0.0001) (Fig. 3D). SEM images of utricular *Myo6*^*sv/sv*^ hair cells revealed changes in the staircase organization of the hair bundle. At P0, hair cells from mutant mice presented a hair bundle with short stereocilia next to a bundle of long stereocilia (Fig. 3E). By P3, *Myo6*^*sv/sv*^ hair bundles showed a complete loss of staircase pattern and displayed a bundle of stereocilia with uniform length (Fig. 3F).

Vestibular-related behavior of *Myo6*^*+/+*^ and *Myo6*^*sv/sv*^ mice was assessed using swim and rotarod tests. Unlike most vestibular behavioral tests, the swim test restricts the animal’s access to visual and proprioceptive modalities, forcing it to rely predominantly on vestibular information, thereby facilitating the detection of vestibular dysfunction even in mice that otherwise maintain normal posture (Gray et al. 1988). During the swim test at P30, *Myo6*^*+/+*^ mice immediately resurfaced and swam for the entire test period (Fig. 4A). In contrast, *Myo6*^*sv/sv*^ mice began spiraling in the water while submerged and had to be quickly rescued, suggesting vestibular dysfunction. In the rotarod test at P30, *Myo6*^*sv/sv*^ mice stayed on the rod for a shorter time compared to *Myo6*^*+/+*^ mice (t(15) = 12.0, *p* < 0.0001; Fig. 4B), suggesting impaired balance. Heterozygous mice displayed a similar phenotype to wild-type mice in both the swim and rotarod tests (Fig. S2).

### Snell’s waltzer mice display hyperactivity

Motor activity of *Myo6*^*+/+*^ and *Myo6*^*sv/sv*^ mice was assessed by a 15 min-long open- field test at P30. *Myo6*^*sv/sv*^ travelled longer distances (mean = 13,353 cm, SD = 4295) than *Myo6*^*+/+*^ mice (mean = 7,136 cm, SD = 1639) (t(20) = 5.0, *p* < 0.0001; Fig. 5A). *Myo6*^*sv/sv*^ also displayed more full-body rotations than *Myo6*^*+/+*^ mice (t(20) = 7.4, *p* < 0.0001; Fig. 5B).

Mice with vestibular dysfunction exhibit a unique spatial exploration strategy in response to a novel environment (Avni et al. 2009). Habituation to a novel space of *Myo6*^*+/+*^ and *Myo6*^*sv/sv*^ mice was assessed by a 60 min-long open-field test at P30. The distance travelled was analyzed by a two-way repeated measures ANOVA with groups (*Myo6*^*+/+*^ *vs. Myo6*^*sv/sv*^) and periods (6 × 10 min) as independent variables. *Myo6*^*sv/sv*^ mice travelled a longer distance (M = 16,676 cm, SD = 4646) compared to *Myo6*^*+/+*^ mice (mean = 3,488 cm, SD = 874.5) (F(1,10) = 31.8, *p* = 0.0002; Fig. 6A), confirming hyperactivity in the mutant mice. *Myo6*^*sv/sv*^ mice showed a progressive increase in activity, while *Myo6*^*+/+*^ mice showed the expected progressive reduction in activity across periods, resulting in a significant group by time interaction (F(5,50) = 7.4, *p* < 0.0001; Fig. 6A). A side effect of *Myo6*^*sv/sv*^ mouse hyperactivity was their almost immediate entrance to the center zone of the open field, while *Myo6*^*+/+*^ mice typically avoided the center zone during the initial period in a novel open field (compare Fig. 6B with Fig. 6D). Subsequently, travel path heatmaps show that *Myo6*^*sv/sv*^ mice, like *Myo6*^*+/+*^, preferred to move along walls of the open field (Fig. 6C, E), before roaming freely around the entire field (Fig. 6F, G).

Lastly, to assess mouse behavior in a familiar environment, we used a PhenoTyper home-cage system for three consecutive days beginning at P30. The distance travelled was analyzed using a three-way repeated measures ANOVA with groups (*Myo6*^*+/+*^ vs. *Myo6*^*sv/sv*^), days (day 1, day 2 and day 3), and daily phase (active vs. inactive) as independent variables. *Myo6*^*sv/sv*^ mice traveled a longer distance compared to *Myo6*^*+/+*^ mice, more during the active than the inactive daily phase (group by daily phase interaction, F(1,14) = 18.9, *p* = 0.0007; Fig. 7), suggesting that hyperactivity is modulated by normal circadian rhythm in *Myo6*^*sv/sv*^ mice. *Myo6*^*sv/sv*^ mice decreased the travelled distance across days more than *Myo6*^*+/+*^ mice (group by day interaction, F(2,28) = 6.2, *p* = 0.006; Fig. 7), suggesting that hyperactivity is modulated by normal long-term habituation to familiar space. Finally, the decrease in traveled distance across days by *Myo6*^*sv/sv*^ relative to *Myo6*^*+/+*^ mice was evident during the active compared to inactive daily phase (group by day by daily phase, (F(2,28) = 4.5, *p* = 0.021), suggesting that hyperactivity is modulated simultaneously by normal circadian and long-term habituation processes.

### Gene replacement in Snell’s waltzer mice failed to rescue auditory and vestibular function

To assess gene replacement therapy in Snell’s waltzer mice, we generated two vectors each with a different promoter. Specifically, we generated AAV2/9-PHP.B-CMV-Myo6 and AAV2/9-PHP.B-CB6-Myo6-HA at similar titers and assessed the vectors in HeLa cells. HeLa cells were successfully transfected with both vectors, resulting in myosin VI expression (Fig. S3). To assess the rescue of auditory and vestibular functions in the mutant, we performed P0 injections in *Myo6*^*sv/sv*^ mice. Two injection routes were used, either through the posterior semi-circular canal or the utricle. The auditory and vestibular rescue of the injected mice was evaluated at P30 by ABR, swim, and rotarod tests. ABR revealed no evidence of hearing recovery (Fig. 8A). In the swim test, mice exhibited spiral swimming and required rescue from the water, indicating no vestibular recovery (Fig. 8B). In the rotarod test, mice remained on the rod for a very short time compared to wild-type controls (*p* < 0.0001 for both groups; Fig. 8C). Immunofluorescence confirmed successful delivery of HA-tagged myosin VI to the hair cells of the cochlea and the utricle (Fig. 8D, E). Furthermore, the percentage of transduced cells was calculated by dividing the number of HA-labeled cells by the total cell count, yielding a transduction rate of 88.7%. Finally, to rule out potential adverse effects from the injections or viral overexpression, wild-type mice were injected with either one of the viruses; no significant differences were observed across tests (Fig S4). Collectively, these findings indicate that Snell’s waltzer mice did not exhibit rescue of auditory or vestibular function upon P0 gene therapy treatment.

## Discussion

Hearing loss and balance impairment are highly prevalent issues with different etiologies (Agrawal et al. 2013; World Health Organization 2025). While both can occur due to environmental factors, such as exposure to loud noise, many pathogenic genetic variants are causally related to these impairments (Carlson et al. 2025; Taiber et al. 2022). The *Myo6* gene is involved in hearing and vestibular function, with different mutations associated with varying degrees of phenotype severity (Avraham et al. 1995; Self et al. 1999). While auditory consequences of *Myo6* mutation have been previously described, balance behavior outcomes have been less studied. In the present study, we analyzed morphological changes in cochlear and vestibular hair cells of Snell’s waltzer mutant mice across developmental stages using confocal and SEM imaging. We also assessed auditory and vestibular behavioral phenotype using ABR, DPOAE, open field, swim, rotarod, and PhenoTyper home-cage monitoring tests. Lastly, we attempted to rescue these phenotypes by gene replacement therapy, injecting AAV expressing *Myo6* in newborn mutant mice.

Myosin VI is essential for maintaining the morphological integrity of cochlear hair cells and auditory function (Avraham et al. 1995; Hertzano et al. 2008; Melchionda et al. 2001). Different mutations in *Myo6* lead to varying impairments of stereocilia formation, uniformly associated with hearing loss (Hertzano et al. 2008; Seki et al. 2017; Wang et al. 2019; Williams et al. 2013; Xue et al. 2022). Here, we report that *Myo6*^*sv/sv*^ cochlear hair cells are affected as early as P0, with progressive deterioration of hair bundles morphology up to P30, resulting in profound hearing loss. This progressive deterioration of cochlear hair cell morphology, beginning with loss of characteristic structure and fusion of the stereocilia, followed by loss of orientation, and culminating in hair cell loss, with the remaining cells exhibiting a single elongated stereocilium, appears consistent with findings reported in the previous Snell’s waltzer study (Self et al. 1999), as well as the fusion in the dominant allele, *Myo6*^*Tlc*^ (Hertzano et al. 2008). Several hypotheses have been proposed to explain the fusion in the stereocilia. One is that myosin VI maintains bundle integrity by anchoring the apical membrane to the cuticular plate (Self et al. 1999) and that the forces exerted on the actin paracrystal and a possibly reduced membrane-actin coupling could result in dysregulated actin treadmilling within stereocilia, ultimately perturbing the staircase architecture of the hair bundle (Hertzano et al. 2008). The extreme lengths of the stereocilia measured in our study suggest that unregulated, active stereocilia elongation, driven by unopposed actin polymerization, may demonstrate that myosin VI not only serves as a static anchor but also as a mechanical ‘brake’ essential for limiting the upward growth of the actin core.

Similar stereocilia elongation has been observed *in Myo7a*^*Hdb*^ and *Clic5*^*680T>*C^ mice (Avni et al. 2009; Hahn et al. 2025). The *Myo6* gene mutation appears to induce hyperactivity (Avraham et al. 1995); however, the resulting impairments in the morphology of the vestibular hair cells and the balance phenotype had not been studied until now. Here, we report that *Myo6*^*sv/sv*^ vestibular hair cells are affected as early as P0, with a progressive deterioration of the hair cells’ morphology continuing up to P30. This change starts similarly to the cochlea, with a loss of staircase structure followed by elongated stereocilia; however, in the utricle, we observe no hair cell loss and a distinct absence of stereocilia fusion. These diverging phenotypes could arise from a difference in stereocilia density and architecture. It has been suggested that the fusion of the stereocilia due to the loss of myosin VI anchoring occurs when neighboring stereocilia touch and zip from bottom to top together and into a single mass (Self et al. 1999). A less dense and more spaced stereocilia in the utricle can allow the elongated stereocilia to persist without the formation of one elongated stereocilia.

Thus, the *Myo6* mutation is associated with a severe morphological impairment of the cochlear and vestibular stereocilia ; however hair cell loss prevails selectively in the cochlea. Myosin VI is part of a molecular complex with other proteins, such as Clic5 , radixin, and taperin, serving as an anchor that stabilizes membrane-actin filament linkages at the base of hair cell stereocilia (Salles et al. 2014). Future studies may reveal that the selective resilience of vestibular hair cells derives from the complex’s lower dependency on myosin VI in vestibular than cochlear hair cells.

As expected, Snell’s waltzer mice displayed profound balance impairments convincingly demonstrated by swim and rotarod tests at P30. In addition to imbalance, Snell’s waltzer, like other inner ear mutants, exhibited profound hyperactivity in the open-field test at P30 (Avni et al. 2009; Hahn et al. 2025). Hyperactivity is considered part of the vestibular “syndrome” as it is observed in compound vestibular and cochlear mutants, but not in selective cochlear mutants (Antoine et al. 2017; Dere et al. 2003). Three features of this vestibular-associated hyperactivity are of interest.

First, Snell’s waltzer hyperactivity features bouts of fast circular locomotion. Circling cannot be explained by asymmetric peripheral or central damage, as bouts of turning are observed in both directions. Spatial constraints should also be excluded, as the circling diameter of Snell’s waltzer mice in the 50 by 50 cm open-field was similar to the circling diameter of Headbanger vestibular mice in the 120 by 120 cm open field (Avni et al. 2009). We consider the possibility that the fast circling represents the mice’s attempt to stimulate the surviving vestibular function. This suggestion is supported by wild-type mice, tested in the microgravity environment of the International Space Station (ISS), showed increased activity and circling compared to ground controls (Ronca et al. 2019).

Second, Snell’s waltzer hyperactivity was observed selectively during the active-dark phase of the circadian rhythm, implying it is well aligned with the environmental cues of the circadian rhythm. The preserved circadian rhythm is surprising since the vestibular mutation should interfere with the suprachiasmatic nucleus (SCN) control of the behavioral circadian rhythm (Horowitz et al. 2004). Indeed, total vestibular loss disrupted the circadian rhythm of core body temperature and locomotory activity in rats. However, the behavioral disruption lasted only a few days and was followed by a progressive recovery of the normal circadian rhythm (Martin et al. 2015). By testing the activity at P30, we likely missed the acute disruption of the circadian rhythm in Snell’s waltzer mice.

Third, Snell’s waltzer hyperactivity is associated with delayed spatial learning. Mouse activity in the open-field test reflects the dynamics of an approach-avoidance conflict, in which the drive to explore is counterbalanced by anxiety-driven avoidance of novel space (La-Vu et al. 2020). Wild-type mice showed a gradual decrease in activity in the 60-min open-field test. This likely reflects the initial rapid dissipation of anxiety, which boosts exploration, followed by the gradual acquisition of spatial familiarity, which in turn lowers exploration. In contrast, Snell’s waltzers showed gradual increase in activity. This likely reflects enhanced anxiety in vestibular mutants (Avni et al. 2009), combined with deficient acquisition of spatial memory and orientation familiarity in vestibular mutants (Avni et al. 2009; Smith et al. 2025). Indeed, Snell’s waltzers showed habituation of the hyperactivity only in the 3-day home cage test.

Finally, we report on the present difficulty to recover hearing and vestibular functions by AAV gene replacement therapy. Gene therapy for human deafness has recently been successful, in the form of AAV gene replacement for otoferlin (*OTOF*) pathogenic variants (Lv et al. 2024; Qi et al. 2025; Wang et al. 2024). Improvements were seen in pure-tone-average hearing levels, indicating that this form of gene therapy is promising in humans (Qi et al. 2025). The optimal therapeutic effect was detected in 5- to 8-year-olds, suggesting that the timing of delivery is critical. Moreover, the advantage of recovering *Otof* is that most inner and outer hair cells, as well as spiral ganglion neurons, remain present after birth in *Otof*-null mice.

Gene therapy for autosomal recessive mutations in *MYO6* may be more challenging, as we demonstrate loss of hair cells already at P0 in the Snell’s waltzer mice and absence of auditory and vestibular recovery with neonatal gene replacement therapy. Late-onset forms of *MYO6-*related deafness may be more amenable to gene therapy. Indeed, gene therapy has been successful for an autosomal dominant *MYO6* model using gene editing (Xue et al. 2022), a mutation associated with degeneration of hair cells around 3 weeks after birth. Earlier, *in utero* gene transfer has been proposed for treating hearing loss (Gubbels et al. 2008; Kong et al. 2024) and may be considered in the future. This strategy may be amenable for the treatment of hearing loss associated with autosomal recessive mutations in *Myo6*, such as in Snell’s waltzer mice.

## Conclusion

In summary, our findings demonstrate that the Snell’s waltzer mutation in the *Myo6* gene results in a congenital morphological phenotype affecting both the cochlear and vestibular systems, with distinct structural alterations contributing to deafness and vestibular impairment, respectively. The data highlight the critical role of the vestibular system as a coordinating mediator of motor function, rather than a primary driver of motor activity. The inability to rescue auditory and vestibular function postnatally highlights the narrow therapeutic window for effective intervention, thus underscoring the challenges gene therapy may face for congenital forms of *MYO6*-related deafness and vestibular impairment.

## Supplementary Information

Below is the link to the electronic supplementary material.


Supplementary Material 1


## Data Availability

No datasets were generated or analyzed during the current study.Supplementary Information The online version contains supplementary material.
