# Supplementary material for: Assessment of auditory and vestibular function and gene therapy in the Snell’s waltzer mouse model of human deafness and balance dysfunction
Source: Mamm Genome. 2026 Aug 28;37(1):99. doi: 10.1007/s00335-026-10263-y (PMC13525007; doi:10.1007/s00335-026-10263-y)
Supplement: Supplementary file 1 [file 335_2026_10263_MOESM1_ESM.pdf]

Supplementary Information

Assessment of auditory and vestibular function and gene therapy in the Snell's waltzer mouse model of human deafness and vestibular dysfunction

Eyal Marton • Roni Hahn • Lior Bikovski • Gwenaëlle S.G. Géléoc • Jeffrey R. Holt • Matti Mintz • Karen B. Avraham

Corresponding author: Karen B. Avraham, karena@tauex.tau.ac.il
Department of Human Genetics & Computational Medicine, Gray Faculty of Medical & Health Sciences and Sagol School of Neuroscience, Tel Aviv University, Tel Aviv 6997801, Israel

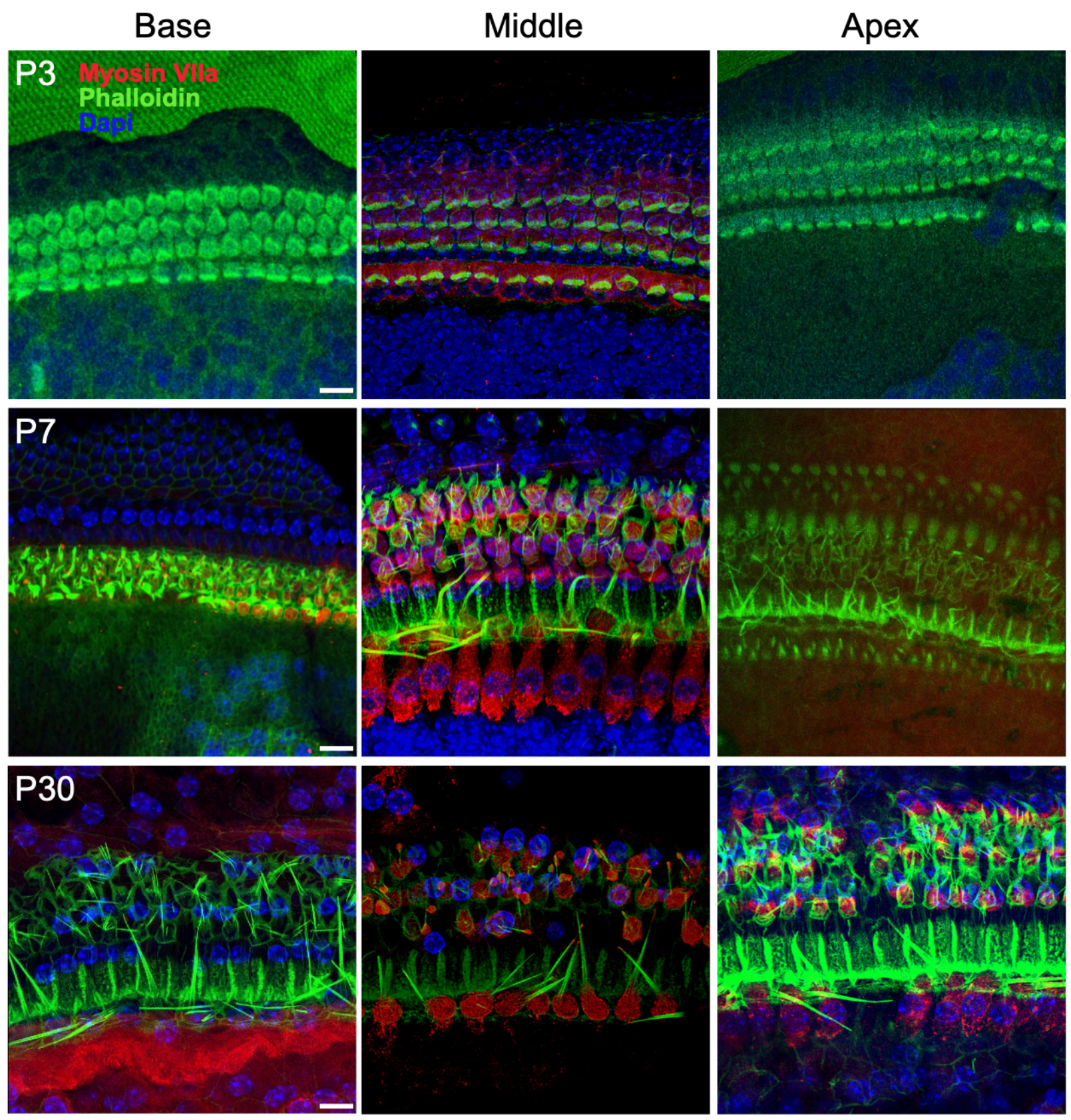

Fig. S1 The changes in *Myo6$^{sv/sv}$* mice were similar across the basal, middle, and apical regions of the cochlea. Representative X2 zoomed confocal images of the different regions of *Myo6$^{sv/sv}$* cochleae labeled with antibodies for hair cell marker myosin VIIa (red), phalloidin (green), and DAPI (blue), at P3, P7, and P30. Scale bars: 10 µm.

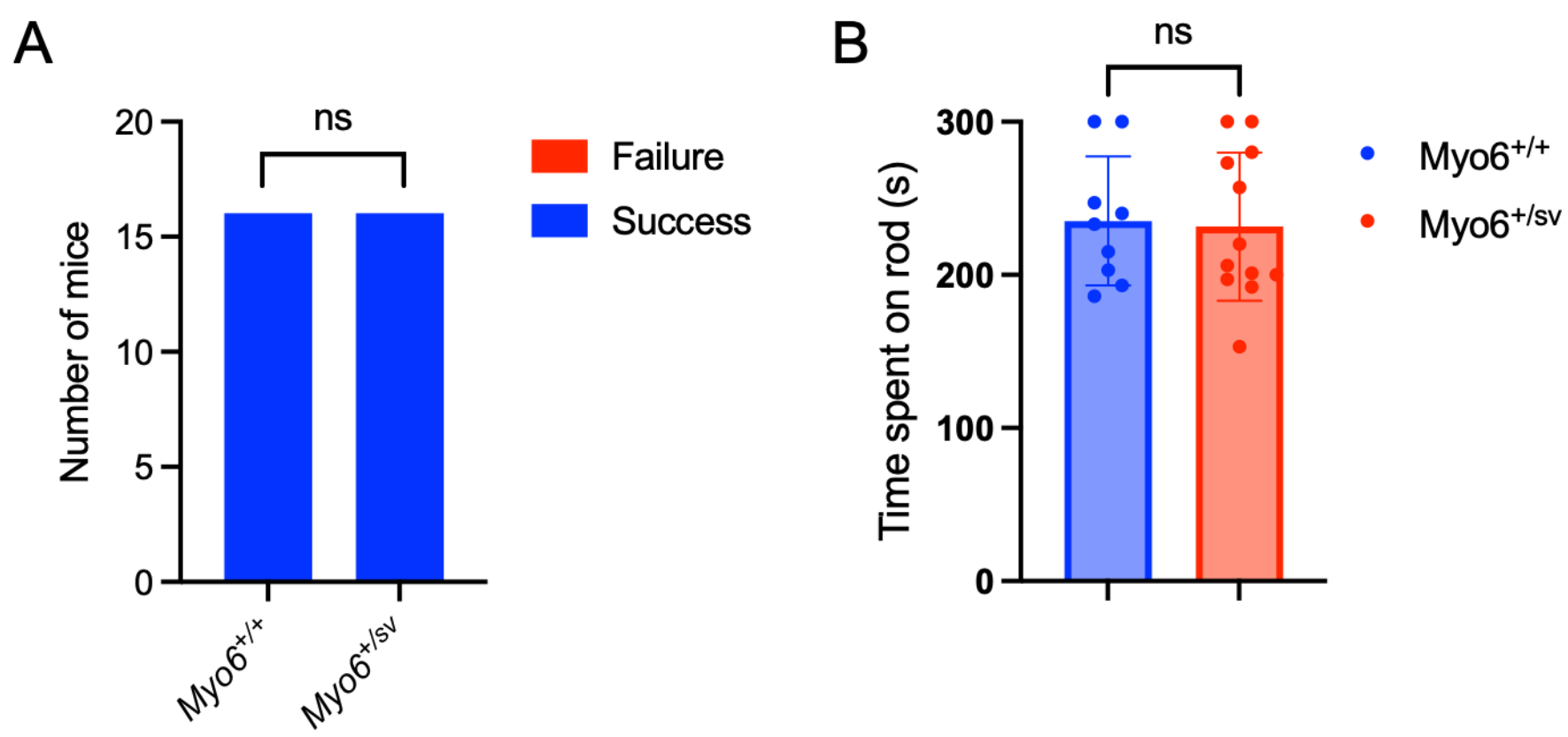

Fig. S2 Behavioral vestibular testing of *sv* heterozygous mice. **A** Swim test performance at of *Myo6*$^{+/+}$ (*n* = 16; males = 8, females = 8) and *Myo6*$^{+/sv}$ (*n* = 16, males = 8, females = 8) mice. **** p < 0.0001. **B** Time on the rotarod (mean ± SD) for *Myo6*$^{+/+}$ (*n* = 16, males = 8, females = 8) and *Myo6*$^{+/sv}$ mice (*n* = 16, males = 8, females = 8). **** p < 0.0001. *n* = number of mice.

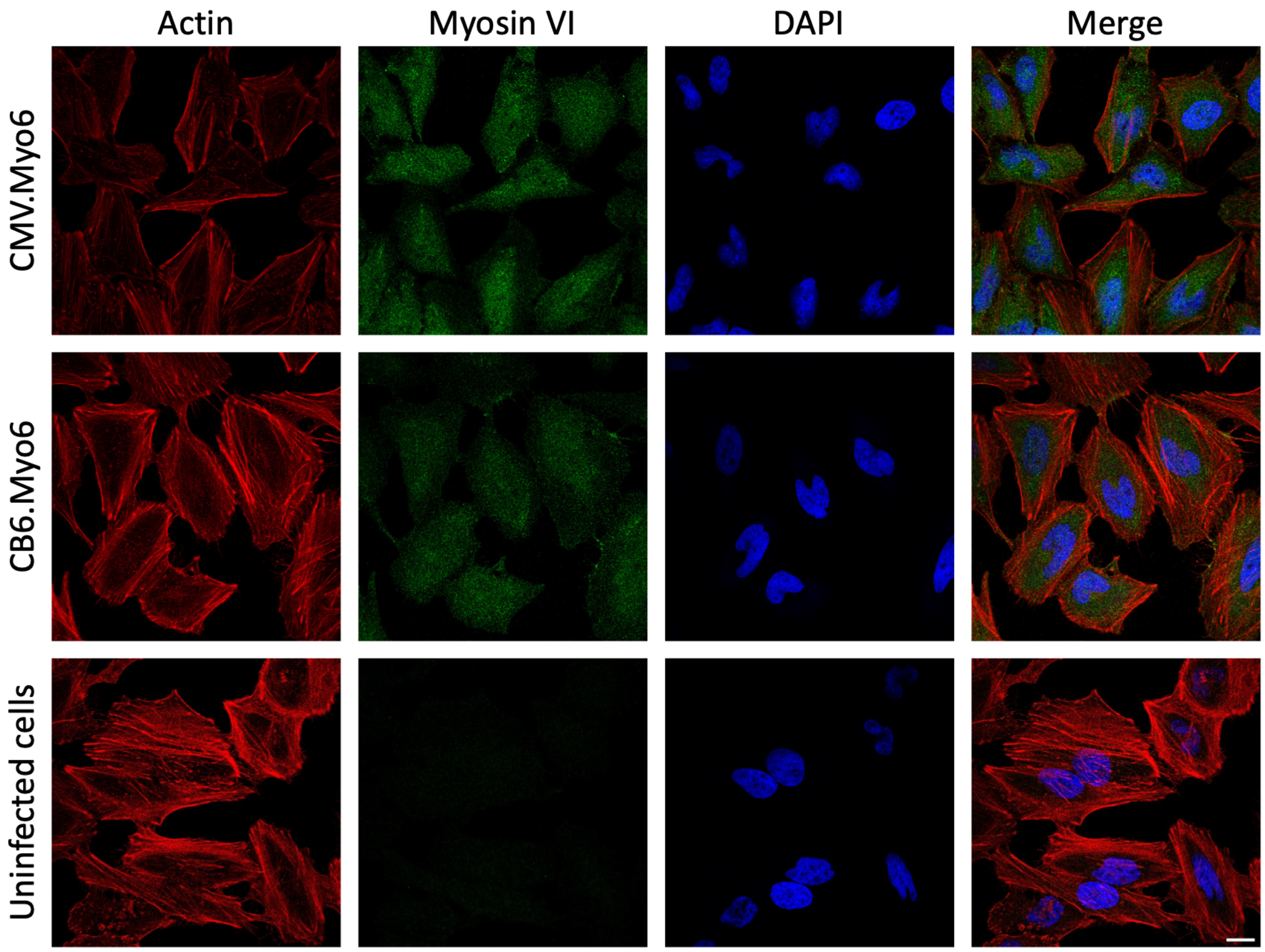

Fig. S3 Myosin VI expression following AAV transduction in HeLa cells. Representative confocal images of HeLa cells transduced with AAV.CMV.Myo6 or AAV.CB6.Myo6, stained with myosin VI (green), actin (red) and DAPI (blue). Secondary antibody-only staining served as a non-immune control. Scale bar: 10 µm.

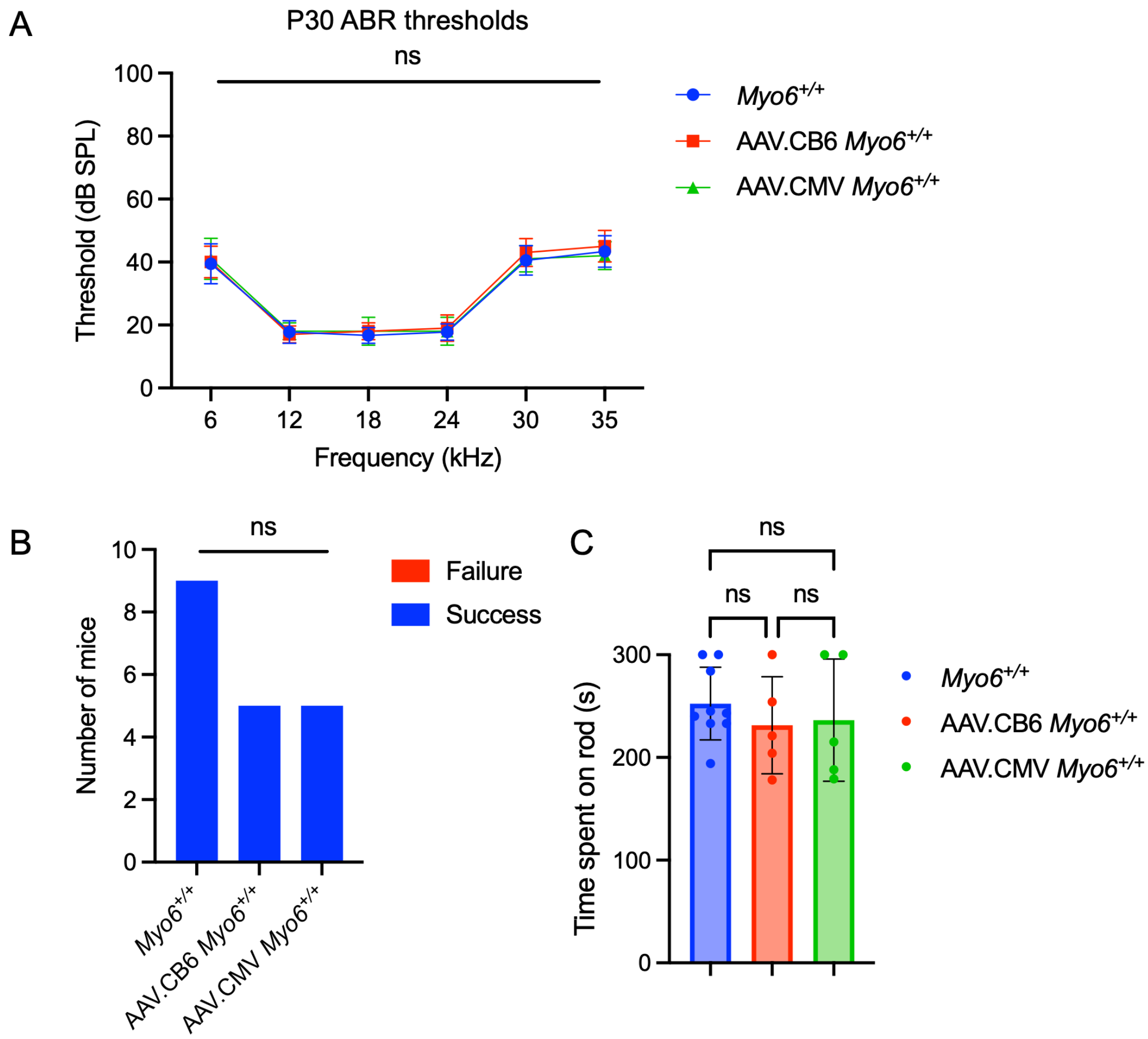

Fig. S4 Wild-type mice were injected with either AAV.CMV.Myo6 or AAV.CB6.Myo6, to rule out potential adverse effects from the injections or viral overexpression. No significant differences were observed across tests. **A** ABR threshold (mean ± SD) at P30 of *Myo6*$^{+/+}$ (*n* = 9; males = 5, females = 4), AAV.CB6 *Myo6*$^{sv/sv}$ (*n* = 5; males = 3, females = 2), AAV.CMV *Myo6*$^{sv/sv}$ (*n* = 5; males = 2, females = 3). **B** Swim test performance at P30 of *Myo6*$^{+/+}$ (*n* = 9; males = 5, females = 4), AAV.CB6 *Myo6*$^{sv/sv}$ (*n* = 5; males = 3, females = 2), AAV.CMV *Myo6*$^{sv/sv}$ (*n* = 5; males = 2, females = 3). **C** Time spent on the rotarod (mean ± SD) at P30 of *Myo6*$^{+/+}$ (*n* = 9; males = 5, females = 4), AAV.CB6 *Myo6*$^{+/+}$ (*n* = 5; males = 3, females = 2) and AAV.CMV *Myo6*$^{sv/sv}$ (*n* = 5; males = 2, females = 3). *n* = number of mice.
